# Computational Insights into Natural Antischistosomal Metabolites as SmHDAC8 Inhibitors: Molecular Docking, ADMET Profiling, and Molecular Dynamics Simulation

**DOI:** 10.3390/metabo13050658

**Published:** 2023-05-15

**Authors:** Abdulrahim A. Alzain, Rua M. Mukhtar, Nihal Abdelmoniem, Fatima A. Elbadwi, Amira Hussien, Elrashied A. E. Garelnabi, Wadah Osman, Asmaa E. Sherif, Amgad I. M. Khedr, Kholoud F. Ghazawi, Waad A. Samman, Sabrin R. M. Ibrahim, Gamal A. Mohamed, Ahmed Ashour

**Affiliations:** 1 Department of Pharmaceutical Chemistry, Faculty of Pharmacy, University of Gezira, Wad Madani 21111, Sudan; ruamubarak1@gmail.com (R.M.M.); nihal.khunaijir@gmail.com (N.A.); fatima.abdelazeem93@gmail.com (F.A.E.); 2 Department of Pharmacology, Faculty of Pharmacy, University of Gezira, Wad Madani 21111, Sudan; amiraph23@gmail.com; 3 Department of Pharmaceutical Chemistry, Faculty of Pharmacy, University of Khartoum, Al-Qasr Avenue, Khartoum 11111, Sudan; rashidgarelnabi@gmail.com; 4 Department of Pharmacognosy, Faculty of Pharmacy, Prince Sattam Bin Abdulaziz University, Al-Kharj 11942, Saudi Arabia; w.osman@psau.edu.sa (W.O.); asmaasherif80@mans.edu.eg (A.E.S.); ahmed.mohamed@psau.edu.sa (A.A.); 5 Department of Pharmacognosy, Faculty of Pharmacy, University of Khartoum, Al-Qasr Avenue, Khartoum 11111, Sudan; 6 Department of Pharmacognosy, Faculty of Pharmacy, Mansoura University, Mansoura 35516, Egypt; 7 Department of Pharmacognosy, Faculty of Pharmacy, Port Said University, Port Said 42526, Egypt; amged.ibrahim@pharm.psu.edu.eg; 8 Clinical Pharmacy Department, College of Pharmacy, Umm Al-Qura University, Makkah 24382, Saudi Arabia; kfghazawi@uqu.edu.sa; 9 Department of Pharmacology and Toxicology, College of Pharmacy, Taibah University, Al-Madinah Al-Munawwarah 30078, Saudi Arabia; wsamman@taibahu.edu.sa; 10 Preparatory Year Program, Department of Chemistry, Batterjee Medical College, Jeddah 21442, Saudi Arabia; sabrin.ibrahim@bmc.edu.sa; 11 Department of Pharmacognosy, Faculty of Pharmacy, Assiut University, Assiut 71526, Egypt; 12 Department of Natural Products and Alternative Medicine, Faculty of Pharmacy, King Abdulaziz University, Jeddah 21589, Saudi Arabia; gahussein@kau.edu.sa

**Keywords:** schistosomiasis, SmHDAC8, allosteric, phytochemicals, molecular docking, molecular dynamics, drug discovery, health and wellbeing

## Abstract

Schistosomiasis is a neglected tropical disease with a significant socioeconomic impact. It is caused by several species of blood trematodes from the genus *Schistosoma*, with *S. mansoni* being the most prevalent. Praziquantel (PZQ) is the only drug available for treatment, but it is vulnerable to drug resistance and ineffective in the juvenile stage. Therefore, identifying new treatments is crucial. SmHDAC8 is a promising therapeutic target, and a new allosteric site was discovered, providing the opportunity for the identification of a new class of inhibitors. In this study, molecular docking was used to screen 13,257 phytochemicals from 80 Saudi medicinal plants for inhibitory activity on the SmHDAC8 allosteric site. Nine compounds with better docking scores than the reference were identified, and four of them (LTS0233470, LTS0020703, LTS0033093, and LTS0028823) exhibited promising results in ADMET analysis and molecular dynamics simulation. These compounds should be further explored experimentally as potential allosteric inhibitors of SmHDAC8.

## 1. Introduction

Schistosomiasis is a neglected tropical disease (NTD) that has a significant socioeconomic impact [1]. The disease is caused by a blood fluke of the genus *Schistosoma*, with *Schistosoma mansoni* having the highest prevalence [2]. It is estimated that schistosomiasis causes 11,792 deaths worldwide each year [3]. While there are three primary species that infect humans, *S. mansoni* is considered a significant public health issue due to its chronic evolution [4]. Unfortunately, there is currently no vaccine available for human use against *S. mansoni* [5]. The sole treatment option is praziquantel (PZQ) monotherapy, which is vulnerable to drug resistance and has limited effectiveness against the juvenile stage [6,7]. Therefore, there is a pressing need for alternative treatment approaches [8].

The histone deacetylase (HDAC) enzymes are attractive targets for developing therapeutics against Schistosoma [7]. HDACs are lysine deacetylases that rely on NAD^+^ or Zn^2+^ and regulate transcription, chromatin structure, gene expression, and several cytoplasmic signaling pathways [9,10,11,12]. Only class I zinc-dependent HDACs, such as SmHDAC1, -3, and -8, and class III HDACs (smSirt1, -2, -5, -6, and -7) have been cloned and described in *S. mansoni* [13,14]. SmHDAC8 is highly expressed throughout the entire life cycle of *S. mansoni*, with its transcripts being more abundant than those of other subtypes [15,16]. Surprisingly, the transcript levels of HDAC8 in human cells are typically lower than those of HDAC1 and HDAC3 [17]. It is worth noting that the human homolog, hHDAC8, is under-expressed in healthy cells [18]. This study focuses on the allosteric site of SmHDAC8, which differs from its human counterpart [7]. Therefore, inhibiting SmHDAC8 may be more selective to *S. mansoni* and have no impact on human host cells.

The diversity and complexity of natural products provide an abundance of potential sources for drug discovery [19]. Traditional medicine (TM) has been explored as an alternative treatment for schistosomiasis, and the investigation of plant extracts has revealed various chemical groups, including steroids, tannins, anthraquinones, glycosides, and terpenoids, which show potential in killing *S. mansoni* worms [20]. Saudi Arabia’s location as a crossroads between three continents has resulted in a vast array of exotic and local medicinal plants. It is estimated that more than 2250 flowering plants are used for traditional medicine in Saudi Arabia, providing a rich source of potential medicinal compounds [21].

In the quest for new and affordable treatments for schistosomiasis, computational approaches have been employed to develop novel schistosomicidal agents [22]. Computer-aided drug design (CADD) techniques, including ligand-based drug design (LBDD) and structure-based drug design (SBDD), such as docking and molecular dynamics (MD), have been utilized in this study due to the need for innovative methods to bring new treatments to patients at a low cost-to-market [23,24]. The aim of this study was to identify potential natural products from Saudi medicinal plants that could inhibit the SmHDAC8 allosteric site using molecular docking, MM-GBSA calculations, ADMET prediction, and molecular dynamics (MD) simulations.

## 2. Methods

The Schrodinger suite was employed for all in silico studies, and Academic Desmond by D. E. Shaw Research was used for molecular dynamics simulations.

### 2.1. Protein Retrieval and Preparation

SmHDAC8 protein structure in complex with its bound ligand (PDB code: 7P2U) was retrieved from the PDB database. Maestro’s Protein Preparation Wizard was utilized to prepare the protein before docking [25]. The structure’s loops and side chains were corrected, and hydrogen atoms were added. At pH 7.4, PROPKA was employed to determine the protonation states of amino acid residues. Finally, the OPLS4 force field was used to optimize the 3D structure of the protein. 

### 2.2. Grid Generation of Protein Receptor

The allosteric site of the SmHDAC8 protein has been determined around the bound ligand using the Receptor Grid Generation tool of Maestro [26]. It uses the coordinates of a ligand that has already formed a complex with the protein to generate a 3D grid with accurate dimensions that represents the receptor’s active area.

### 2.3. Ligands Preparation

A library of 13,257 natural products from 80 Saudi medicinal plants was collected from the LOTUS database and prepared using the MacroModel module of Maestro. The ligand preparation process includes the generation of a maximum of 32 conformations for each ligand. OPLS4 was utilized to minimize the energies of each fragment. 

### 2.4. Molecular Docking and MM-GBSA Binding Free Energy Calculation

The molecular docking was carried out using the Glide docking panel of Maestro. The prepared molecules were subjected to two levels of docking filters, including high throughput virtual screening (HTVS) and extra precision (XP). The top resultant compounds were submitted to Prime of Maestro. During the docking experiment, XP descriptors were created to collect atom-level energy terms such as electrostatic contact, hydrogen bond interaction, pi–pi stacking, and hydrophobic enclosure. 

MM-GBSA binding free energy was calculated using Prime of Maestro [26,27] utilizing the optimum docking poses. Binding free energy calculations are more accurate than docking as it considers the solvation effect. 

### 2.5. In Silico ADME Prediction

Any small molecule must meet ADME properties to be presumed a viable therapeutic candidate. Investigation of physicochemical properties has been carried out on the top-scored ligands using a web server called pkCSM tool. 

### 2.6. Molecular Dynamics (MD) Simulations

Molecular dynamics (MD) simulations were used to examine the stability of docked protein–lead molecule complexes. The MD investigations for the SmHDAC8 protein complexed with the top XP dock scoring compounds were performed for 100 ns using Academic Desmond v6.5, as previously described [28,29]. The energy minimization was performed OPLS3e force field. To easily fulfill the minimal image convention, these complexes were submerged in an orthorhombic box of TIP3P solvent molecules with a size of 10 Å on each side. By adding Na^+^ and Cl^−^ ions, the complexes were neutralized. The maximum number of iterations was set to 2000 during the minimization, and the convergence rate was set at 1.0 kcal/mol. The simulation was conducted in NPT ensemble mode at (300 K) temperature and (1 bar) pressure to achieve a completely converged system for 100 production runs. The particle mesh Ewald was utilized to manage the long-range electrostatics, with a relative tolerance between long and short-range energies of 1 × 10^−9^. A real-space cut-off of 9 was used to analyze short-range interactions. Through the simulations, 1000 frames were gathered for each system. After structure stabilization, the final models in all three complexes were derived by averaging snapshots from the trajectory generated by MD simulations. For the chosen ligand–protein complex, the root mean square deviation (RMSD) was computed. The average change in the location of the selected atoms in a compound in relation to the reference trajectory frame is shown by RMSD.

## 3. Results

**Figure 1:**
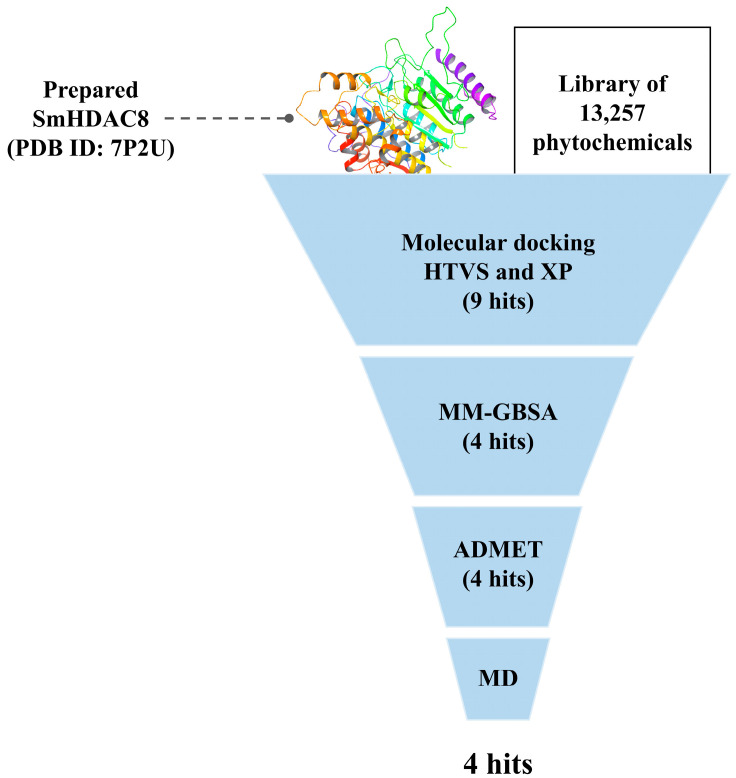
The overall workflow of the study.

The results of the present work are summarized in Figure 1.

### 3.1. Molecular Docking and Free-Binding Energy Prediction

The molecular docking process and free-binding energy prediction in this study proceeded as follows: Firstly, a library of 13,257 phytochemicals was screened against the SmHDAC8 allosteric site to identify potential novel SmHDAC8 lead candidates. The docking procedure was validated by redocking the co-crystalized ligand and comparing it with the estimated redocked pose. The root mean square deviation (RMSD) value obtained was 2.6831 Å, confirming the procedure’s validity. Next, the phytochemicals’ library was docked into the SmHDAC8 allosteric site’s grid using Glide’s HTVS mode, which resulted in 643 compounds with docking scores < −5. The 643 compounds were then subjected to XP docking, and nine compounds with docking scores ranging from −10.782 to −7.054 kcal/mol were shortlisted for this study (Table 1). Notably, the top-ranked nine compounds’ docking scores were superior to the co-crystalized ligand’s score (−5.441 kcal/mol), which was used as a reference to evaluate the results.

Furthermore, the top-ranked nine compounds underwent free-binding energy prediction using the MM-GBSA method. These compounds achieved values ranging from −56.02 to −28.38 kcal/mol, whereas the reference estimated free-binding energy was −53.16 kcal/mol (Table 1).

To achieve the objective of identifying potential SmHDAC8 allosteric inhibitors, we selected the top four compounds based on their MM-GBSA dG binding energies, which were better than the reference. These four compounds, referred to as compounds **1**, **2**, **3**, and **4** in this study, were further analyzed to gain insights into their interactions with the SmHDAC8 allosteric site.

**Figure 2:**
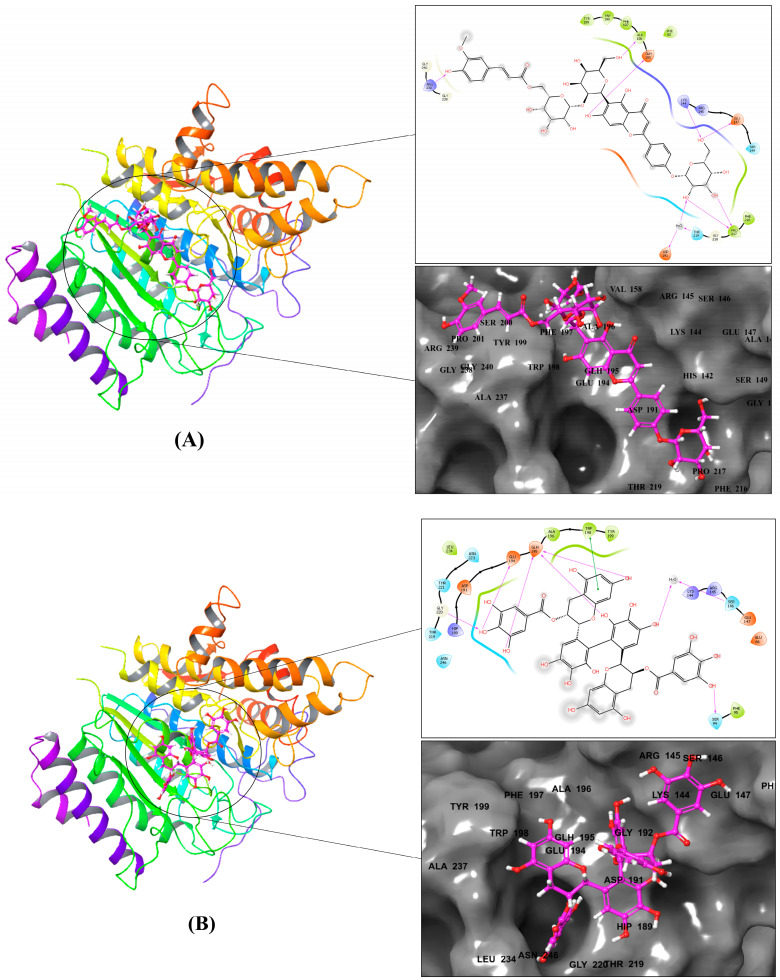

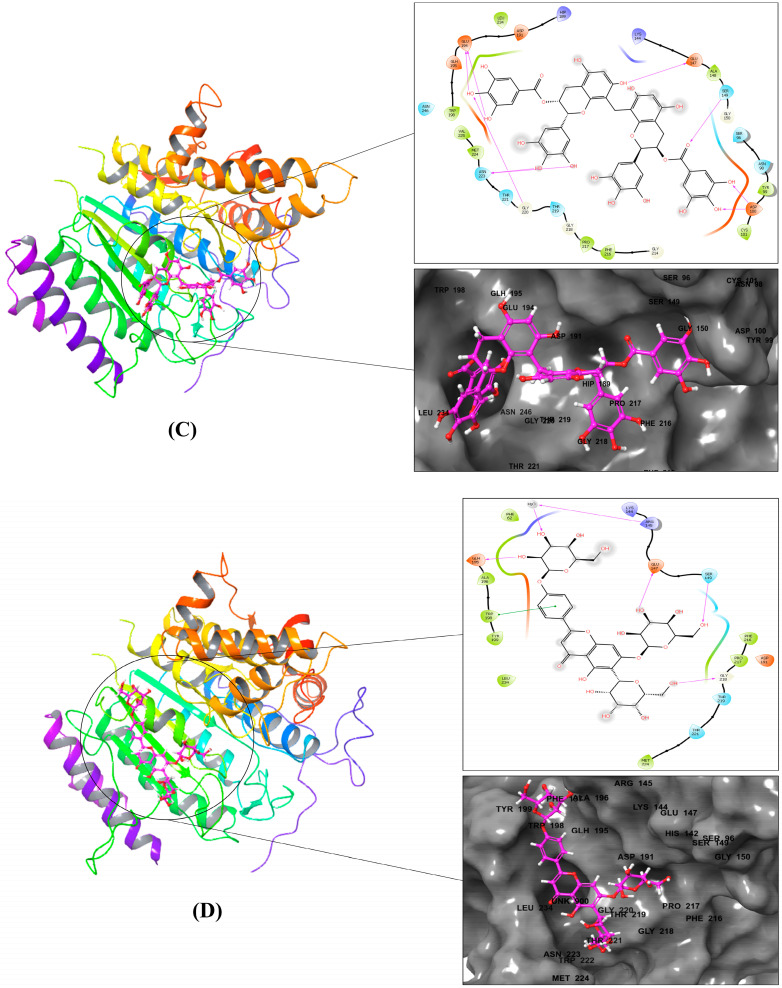
The 2D and 3D diagram of the top compounds with the SmHDAC8 (PDB ID: 7P2U). (**A**) Compound **1**, (**B**) compound **2**, (**C**) compound **3**, and (**D**) compound **4**.

Compound **1** displayed eight hydrogen bond interactions with LYS144, GLU147, ASP191, GLH195, ALA196, PRO217, THR219, and ARG239, as well as seven hydrophobic contacts with PHE62, ALA196, PHE197, TRP198, TYR199, PHE216, and PRO217 (see Figure 2A and Table 2).

In Figure 2B and Table 2, Compound **2** exhibited one pi–pi interaction with TRP198, five H-bond interactions with SER94, SER146, GLU194, GLH195, and GLY220, and five hydrophobic contacts with PHE95, ALA196, TRP198, TYR199, and LEU234. Likewise, in Figure 2C and Table 2, Compound **3** displayed six H-bond interactions with ASP100, GLU147, SER149, GLU194, GLY220, and ASN223, and nine hydrophobic contacts with TYR99, CYS101, ALA148, TRP198, PHE216, PRO217, MET224, VAL225, and LEU234. Lastly, in Figure 2D and Table 2, Compound **4** showed one pi–pi interaction with TRP198, five H-bond interactions with ARG145, GLU147, SER149, GLH195, and GLY218, and eight hydrophobic contacts with PHE62, ALA196, TRP198, TYR199, PHE216, PRO217, MET224, and LEU234.

### 3.2. ADMET Prediction

To predict the ADMET properties of compounds **1**–**4** and the reference, we utilized the pkCSM web server, and the results are presented in Table 3. Firstly, with regard to absorption properties, all four compounds and the reference achieved water solubility (logS) of −2.892 mol/L. Secondly, concerning distribution properties, the logBB values for compounds **1**–**4** and the reference were −3.828, −4.312, −3.417, 0.869, and −0.273, respectively. A compound with a logBB value greater than 0.3 can readily cross the blood-brain barrier, whereas a compound with a logBB value less than -1 is poorly distributed to the brain. Thirdly, in terms of metabolism, none of the four compounds were found to be substrates or inhibitors of CYP3A4, CYP1A2, CYP2C9, and/or CYP2D6. However, the reference was found to be an inhibitor of CYP1A2. Fourthly, concerning excretion, only compound **2** was identified as a renal OCT2 substrate. Finally, with respect to toxicity, none of the four compounds showed AMES toxicity or hepatotoxicity. In contrast, the reference showed AMES toxicity.

### 3.3. MD Simulation

**Figure 3:**
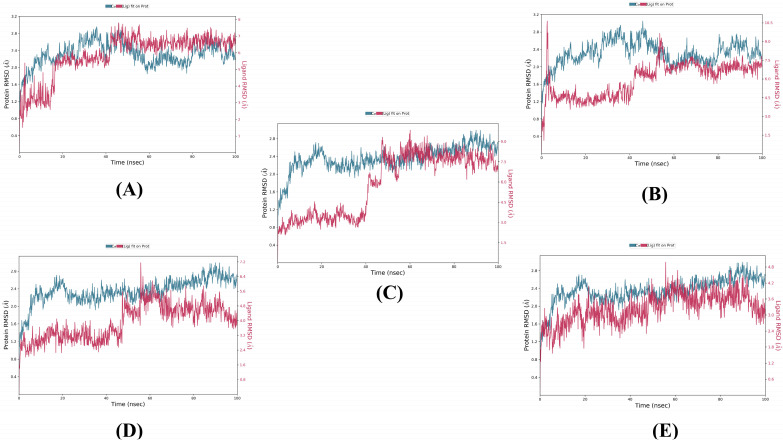
RMSD plots of the top compounds and the reference with the SmHDAC8 (PDB ID: 7P2U) during 100 ns molecular dynamics simulation. (**A**) compound **1**, (**B**) compound **2**, (**C**) compound **3**, (**D**) compound **4**, and (**E**) reference.

The MD simulations were performed for compounds **1**–**4** and the reference in the allosteric site of SmHDAC8 protein for 100 ns. Figure 3 shows the RMSD plots, indicating that the protein underwent similar Cα deviations with an RMSD range of 0.4–3.2 Å and an average value of 2.311992 Å when complexed with compounds **1** and **2**. In contrast, with compounds **3**, **4**, and the reference, the Cα atoms behaved similarly to each other but different from compounds **1** and **2**, with an RMSD range of 0.4–2.8 Å and an average value of 2.350006 Å. An RMSD value of 1–3 Å is generally acceptable for small globular proteins, indicating acceptable stability between the target and the compounds. 

The ligand RMSD ranges were found to be compound **2** (1.5–10.5 Å) > compound **3** (1.5–9 Å) > compound **1** (1–8 Å) > compound **4** (0.8–7.2 Å) > the reference (0.6–4.8 Å). The higher difference in RMSD values between the reference and the three compounds could be due to the larger number of rotatable bonds between the reference (6 bonds) and compounds **1**–**4** (27, 25, 26, and 22 bonds, respectively).

Analyzing both protein and ligand RMSD from the RMSD plots, it can be estimated that compound **3** is the most stable among the four compounds, reaching equilibrium at around 50 ns and maintaining stability with minimum fluctuations. Compound **1** comes next in terms of stability, reaching equilibrium at around 15 ns but undergoing higher fluctuations for the rest of the simulation time. Compounds **2** and **4** behaved similarly during the first 70 ns of the simulation time, reaching equilibrium at around 50 ns, but compound **4** maintained this equilibrium only until 70 ns, while compound **2** maintained it until 80 ns and then underwent fluctuations before returning to the equilibrium state near 100 ns. The reference achieved equilibrium from the beginning of the simulation but experienced high fluctuations throughout the simulation time.

**Figure 4:**
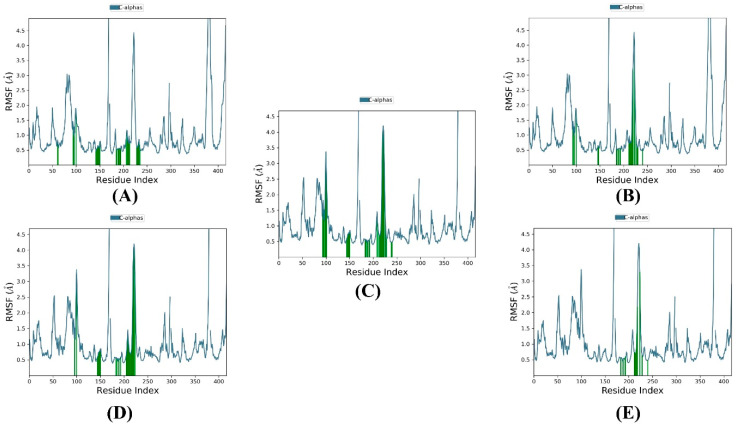
RMSF plots of the top compounds and the reference with the SmHDAC8 (PDB ID: 7P2U) during 100 ns molecular dynamics simulation. (**A**) Compound **1**, (**B**) compound **2**, (**C**) compound **3**, (**D**) compound **4**, and (**E**) reference. Protein residues that interact with the ligand are marked with green-colored vertical bars.

The root mean square fluctuation (RMSF) was computed for each residue within the SmHDAC8 protein in complex with compounds **1**–**4** and the reference. Figure 4 shows two different RMSF patterns in the protein: one shared between compounds **1** and **2** and the other shared between compounds **3** and **4** and the reference complexes. This resulted in two different average RMSF values of 1.035174 Å and 0.987179 Å for the first and second patterns, respectively. The low RMSF values indicate less variability in the protein structure.

**Figure 5:**
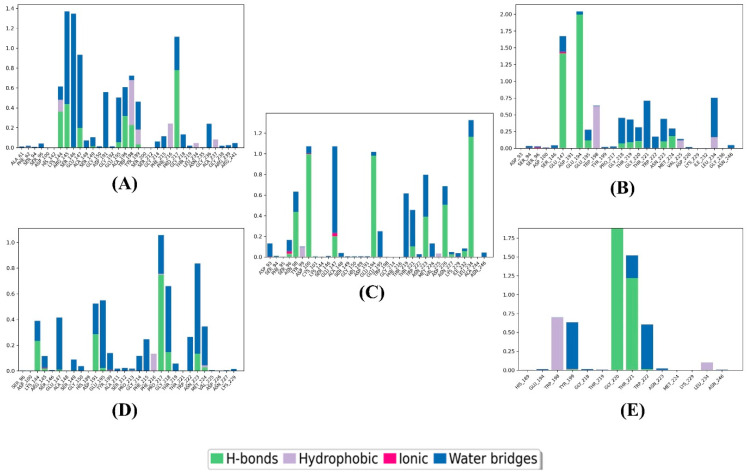
Interactions of the top compounds and the reference with the SmHDAC8 (PDB ID: 7P2U) during 100 ns molecular dynamics simulation. (**A**) Compound **1**, (**B**) compound **2**, (**C**) compound **3**, (**D**) compound **4**, and (**E**) reference.

Figure 5, the interaction analysis histogram, was used to record the binding and non-binding interactions that occurred during the simulation process between the four compounds and the reference with the protein. Compound **1** formed interactions with LYS144 (48% H-bond, 12% hydrophobic, and 10% water bridge), ARG145 (45% H-bond and 93% water bridge), SER146 (136% water bridge), GLU147 (20% H-bond and 70% water bridge), ASP191 (57% water bridge), GLU195 (9% H-bond and 41% water bridge), ALA196 (37% H-bond and 23% water bridge), TRP198 (22% H-bond, 47% hydrophobic, and 4% water bridge), TYR199 (4% H-bond, 16% hydrophobic, and 23% water bridge), PHE216 (22% hydrophobic), Pro217 (80% H-bond and 38% water bridge), and GLY236 (23% water bridge). It should be noted that some residues had interaction values exceeding 100%, which is attributed to these residues forming multiple contacts of the same interaction type with the ligand.

Compound **2** made contact with GLU147 (145% H-bond, 4% ionic, and 25% water bridge), GLU194 (200% H-bond and 5% water bridge), GLU195 (13% H-bond and 15% water bridge), TRP198 (70% hydrophobic and 1% water bridge), GLU218 (9% H-bond and 45% water bridge), THR219 (10% H-bond and 43% water bridge), GLY220 (11% H-bond and 15% water bridge), THR221 (75% water bridge), TRP222 (22% water bridge), ASN223 (11% H-bond and 34% water bridge), MET224 (24% H-bond and 11% water bridge), LEU234 (24% hydrophobic and 2% water bridge), and LEU234 (24% hydrophobic and 78% water bridge).

Compound **3** showed interactions with ASP93 (15% water bridge), SER96 (3% H-bond, 2% ionic, and 13% water bridge), ASN98 (44% H-bond and 18% water bridge), ASP100 (100% H-bond, 1% ionic, and 5% water bridge), GLU147 (20% H-bond, 3% ionic, and 80% water bridge), GLU194 (99% H-bond and 3% water bridge), GLU195 (25% water bridge), THR219 (25% water bridge), THR221 (10% H-bond and 40% water bridge), ASN223 (42% H-bond and 40% water bridge), MET224 (15% water bridge), ASP226 (57% H-bond and 17% water bridge), and LEU234 (120% H-bond and 17% water bridge). 

Compound **4** exhibited interactions with LYS144 (23% H-bond and 17% water bridge), GLU147 (1% H-bond 40% water bridge), ASP191 (30% H-bond and 20% water bridge), GLU195 (2% H-bond and 45% water bridge), PHE215 (24% water bridge), PRO217 (88% H-bond and 25% water bridge), GLY218 (18% H-bond and 45% water bridge), TRP222 (15% water bridge), ASN223 (15% H-bond and 70% water bridge), and MET224 (3% H-bond, 2% hydrophobic, and 33%). 

At last, the reference interacted with TRP198 (72% hydrophobic), TYR199 (1% H-bond and 63% water bridge), GLY220 (185% H-bond), THR221 (125% H-bond and 25% water bridge), and TRP222 (1% H-bond and 60% water bridge).

## 4. Discussion

The urgent need for new drugs to treat eukaryotic parasitic infections, particularly neglected ones that lack effective vaccines and treatment options, is well recognized [30]. One such neglected parasitic infection is schistosomiasis, which afflicts hundreds of millions of people and claims thousands of lives each year in affected areas [31]. Presently, PZQ is the sole available treatment for schistosomiasis, but its use for both treatment and disease control leaves it vulnerable to drug resistance. Moreover, PZQ has a significant limitation in that it is ineffective against the parasitic larval stages, which can lead to treatment failure [32]. Consequently, our study aimed to identify new potential hits for Schistosoma treatment, with a focus on *S. mansoni*, the most widely distributed species [8]. To accomplish this objective, selecting a suitable therapeutic target was critical, and we identified SmHDAC8 as a promising candidate. Experimentally reducing SmHDAC8 expression led to a marked decrease in parasite viability and fertility, and this protein is highly expressed throughout the Schistosoma life cycle, while its human counterpart is poorly expressed in healthy human tissues [33]. Saccoccia et al. made an intriguing discovery when they identified a new allosteric site on the surface of SmHDAC8 near TRP198 using X-ray crystallography. Their findings indicated that two of their compounds, NF2883 and NF2889, bound to a site other than the active site and were likely to inhibit the enzyme in a mixed-type/allosteric manner. Surprisingly, these two compounds showed lower IC_50_ values on SmHDAC8 than the compounds that bound to the active site only. The research team suggested that the binding of these inhibitors to the novel site may lead to an allosteric structural transition. The new allosteric site comprises amino acids from helices α9 and the two loops between β5 and α9 and β5 and β6, respectively [7]. Given the potential for achieving greater selectivity against other human HDAC isoforms and zinc-dependent proteins, we aimed to be among the first groups to exploit this site in drug discovery.

To initiate our molecular modeling work, we docked a library of 13,257 natural compounds from 80 Saudi medicinal plants onto the SmHDAC8 allosteric site using the Glide module of Schrödinger. Glide offers three docking methodologies, namely high throughput virtual screening (HTVS), standard precision (SP), and extra precision (XP). These three methodologies differ in accuracy and time consumption in compound screening, as they employ different scoring functions [21]. HTVS is the least accurate and fastest, reducing the number of intermediate conformations, final torsion refinement, and sampling. XP is the most accurate and slowest, utilizing extensive sampling and a sophisticated scoring function that eliminates false positive results and penalizes compounds with reduced complementarity with the target’s active site [34]. Both HTVS and XP dockings of the library resulted in nine compounds with higher docking scores than the reference (the co-crystallized ligand of the 7P2S structure, NF2889), indicating that these compounds have a higher affinity for the allosteric site than the reference. After performing free-binding energy predictions, four of these compounds exhibited higher dG bind energies than NF2889, suggesting that these compounds are more stable on the allosteric site than the reference. The analysis of the interaction patterns revealed various types of contacts between the best four compounds and the protein, many of which corresponded with Saccoccia et al.’s results [7], the group that identified the allosteric site and was the only one to study compound interactions within it thus far. Saccoccia et al. investigated the interaction pattern of NF2889 and concluded that Trp198 and Glu195 have the highest contribution to the inhibitor binding.

Compounds **2** and **4** were found to exhibit pi–pi stacking interactions with Trp198, while compounds **1**, **2**, and **4** interacted via H-bonds with Glu195. Additionally, Saccoccia et al. reported that NF2889 interacted with GLU194, GLY220, THR221, and Leu234. Compounds **2** and **3** were found to form H-bonds with GLU194 and GLY220, while compounds **2**, **3**, and **4** had a hydrophobic interaction with Leu234 and a polar interaction with THR221. Based on these findings, it was assumed that compound **2** was similar to NF2889 in terms of interaction patterns since it exhibited interactions with all the residues known to interact with NF2889. It is worth noting that compounds **1**–**4** showed a minimum of five interacted residues via H-bond and hydrophobic contact, with a maximum of eight and nine residues, respectively. This justifies their better docking scores, dG bind energies, and potentially higher inhibition activity concerning NF2889. Docking on the allosteric site revealed that NF2889 interacted with four residues via H-bond and three residues via hydrophobic contact. The docking results, free-binding energy prediction, and interaction pattern analysis all suggest that compounds **1**–**4** have a high probability of being better allosteric inhibitors of SmHDAC8 than NF2889.

Furthermore, compounds **1**–**4** were subjected to in silico ADMET prediction using the pkCSM tool to assess their pharmacokinetics properties and toxicological profiles. Compound **4** was found to have a logBB value of 0.869, indicating that it can readily cross the blood-brain barrier (BBB). This property could be useful in treating CNS schistosomiasis that occasionally occurs with *S. mansoni* infection [35]. However, none of the four compounds showed interference with metabolism or positive toxicity. In contrast, NF2889 was found to be an inhibitor of CYP1A2 and had a positive AMES result, suggesting potential drug-drug interactions and mutagenicity [36].

Finally, molecular dynamics (MD) simulation was utilized to investigate the stability, flexibility, and binding and non-binding interactions of the screened compounds–protein complexes. Unlike previous techniques that treated the protein and ligands as rigid molecules, MD simulations took into account the conformational changes of the receptor and ligand, mimicking the real scenario in human body conditions [37,38,39]. The low RMSD and RMSF values observed suggest less flexibility of the protein structure and suitable stability of the screened compounds–protein complexes.

A literature review was conducted to gain a deep insight into the identity of the top four compounds. Compounds **1**–**4** were identified as flavonoids, which belong to a class of secondary metabolites that contain polyphenol rings in their structures and are widely distributed throughout the plant kingdom [40]. Flavonoids can be found in various plant parts where they serve important functions in growth and defense and are also responsible for the attractive color, odor, and flavor of plants to pollinators [41]. Based on the position of substitutions and the degree of saturation and oxidation of the flavonoid nucleus (flavone), they can be classified into several groups, such as flavones, flavonols, catechins, and chalcones, which can be further divided into subgroups [42]. Flavonoids are known for their antioxidant, anti-inflammatory, anti-mutagenic, and anti-carcinogenic properties and have been identified as potent inhibitors of many enzymes, making them an attractive focus for the pharmaceutical industry [41].

Compound **1**, isovitexin 2′′-(6′′′-(E)-feruloylglucoside) 4′-glucoside, was first isolated and structurally characterized from the leaves of *Cucumis sativus* [43]. Compound **4**, saponarin 4′-O-glucoside, was initially discovered in a flowering herb of *Lagenaria siceraria* [44], but *Cucumis sativus* is now the primary known source of this compound [45]. Interestingly, both compounds **1** and **4** are apigenin glycosides, with isovitexin (apigenin 6-C-glucoside) serving as the precursor for the biosynthesis of saponarin (apigenin 6-C-glucoside-7-O-glucoside) [46]. Apigenin is a significant subgroup of flavones and is commonly found in foods such as parsley, chamomile, oregano, celery, vine spinach, and artichokes [47].

Compounds **2** and **3** are known as Theasinensin A and Oolonghomobisflavan A, respectively. Theasinensin A was first identified in green tea, while Oolonghomobisflavan A was detected in Oolong tea [48,49]. Both teas come from the *Camellia sinensis* tree. Notably, Theasinensin A and Oolonghomobisflavan A are both (-)-epigallocatechin-3-gallate (EGCG) dimers, but they differ in the way the two EGCG monomers are connected. Theasinensin A connects the B rings through a C-C link at the 2,2′ position, while Oolonghomobisflavan A connects the A rings via a methylene bridge at the 8,8′ position.

Regarding biological activity, compounds **1** and **4** do not have any recorded activity. However, compounds **2** and **3** have several citations in various research topics, including coronavirus. *Camellia sinensis* extracts are known for their anti-amoebic activity [50,51,52], and catechins are promoted as emerging anti-parasitic agents [53]. Furthermore, a study by Paveto et al. suggested EGCG as an anti-*Trypanosoma cruzi* agent [54]. In the context of this study, our findings contribute to the existing research on the anti-parasitic activity of Camellia sinensis extracts by reporting the potential anti-schistosomiasis activity of two of its components: Theasinensin A (compound **2**) and Oolonghomobisflavan A (compound **3**). Additionally, we are the first group to report that two compounds from Cucumis sativus (compounds **1** and **4**) have anti-parasitic activity, specifically anti-schistosomiasis.

## 5. Conclusions

To identify potential allosteric SmHDAC8 inhibitors, a library of 13,257 compounds underwent several molecular modeling approaches, including molecular docking, MM-GBSA calculations, ADMET analysis, and MD simulation. Following molecular docking and MM-GBSA calculations, four compounds (compounds **1**–**4**) exhibited better docking scores and MM-GBSA dG binding energies than the reference, suggesting they have the potential to be more potent inhibitors of the SmHDAC8 allosteric site. The common interactions between these compounds and the reference, in addition to other interactions, supported this hypothesis. Furthermore, ADMET analysis indicated promising results concerning the pharmacokinetics and toxicological profile of these compounds. The MD simulation further confirmed the stability of the compound–receptor complexes. These results could contribute to the development of allosteric inhibitors for the treatment of schistosomiasis, and further evaluation of compounds **1**–**4** is recommended.

## Figures and Tables

**Table 1:** The top-ranked nine compounds according to the docking score and MM-GBSA dG binding energy on the SmHDAC8 allosteric site.

Lotus ID	Docking Score	MM-GBSA dG Binding Energy
LTS0233470 (compound **1**)	−10.782	−55.14
LTS0020703 (compound **2**)	−10.375	−55.69
LTS0033093 (compound **3**)	−10.114	−56.02
LTS0028823 (compound **4**)	−8.046	−54.66
LTS0172554	−8.021	−42.4
LTS0270922	−7.627	−28.38
LTS0108335	−7.292	−30.11
LTS0146028	−7.271	−33.35
LTS0031359	−7.054	−31.26
Co-crystalized ligand (PDB ID 7P2U)	−5.441	−53.16

**Table 2:** Interacted residues of the SmHDAC8 allosteric site with the best four compounds.

Compound	Pi–pi Stacking Interaction	H-Bond Interaction	Hydrophobic Interaction	Other Interactions
**1**	-	LYS144, GLU147, ASP191, GLH195, ALA196, PRO217, THR219, ARG239	PHE62, ALA196, PHE197, TRP198, TYR199, PHE216, PRO217	Charged (positive): ARG139, LYS144, ARG145 Charged (negative): GLU147, ASP191, GLH195 Polar: SER149, THR219
**2**	TRP198	SER94, SER146, GLU194, GLH195, GLY220	PHE95, ALA196, TRP198, TYR199, LEU234	Charged (positive): LYS144, ARG145, HIP189 Charged (negative): GLU66, GLU147, ASP191, GLU194, GLH195 Polar: SER94, SER146, THR219, THR221, ASN223, ASN246
**3**	-	ASP100, GLU147, SER149, GLU194, GLY220, ASN223	TYR99, CYS101, ALA148, TRP198, PHE216, PRO217, MET224, VAL225, LEU234	Charged (positive): LYS144, HIP189 Charged (negative): ASP100, GLU147, ASP191, GLU194, GLH195 Polar: SER96, ASN98, SER149, THR219, THR221, ASN223, ASN246
**4**	TRP198	ARG145, GLU147, SER149, GLH195, GLY218	PHE62, ALA196, TRP198, TYR199, PHE216, PRO217, MET224, LEU234	Charged (positive): LYS144, ARG145 Charged (negative): GLU147, ASP191, GLH195 Polar: SER149, THR219, THR221

**Table 3:** The ADMET properties of the best four compounds.

Compound	Absorption	Distribution	Metabolism						Excretion	Toxicity	
			CYP								
			2D6	3A4	2D6	3A4	1A2	2C9			
	Water Solubility	Blood-Brain Barrier Permeability	Substrate		Inhibitor				Renal OCT2 Substrate	AMES Toxicity	Hepatotoxicity
	Numeric (log mol/L)	Numeric (log BB)	Categorical (Yes/No)						Categorical (Yes/No)	Categorical (Yes/No)	
**1**	−2.892	−3.828	No	No	No	No	No	No	No	No	No
**2**	−2.892	−4.312	No	No	No	No	No	No	Yes	No	No
**3**	−2.892	−3.417	No	No	No	No	No	No	No	No	No
**4**	−2.892	0.869	No	No	No	No	No	No	No	No	No
Reference	−2.892	−0.273	No	No	No	No	Yes	No	No	Yes	No

## Data Availability

The datasets generated during and/or analyzed during the current study are available from the corresponding author upon reasonable request.
